# EGFR-Targeted Therapies: A Literature Review

**DOI:** 10.3390/jcm13216391

**Published:** 2024-10-25

**Authors:** Calista Sha, Paul C. Lee

**Affiliations:** Department of Cardiothoracic Surgery, Long Island Jewish Medical Center, New Hyde Park, NY 11040, USA; csha@northwell.edu

**Keywords:** lung cancer, NSCLC, EGFR, TKI

## Abstract

Lung cancer remains the leading cause of cancer death in the United States, underscoring the critical need to optimize treatment strategies. Compared to conventional treatments such as surgical resection, radiotherapy, chemotherapy, and immunotherapy, targeted therapy stands out for its higher selectivity and minimal adverse effects. Among these, epidermal growth factor receptor (EGFR) tyrosine kinase inhibitors (TKIs) are the most widely used in targeted therapy for non-small-cell lung cancer (NSCLC). In our paper, we will conduct a comprehensive review of current literature on EGFR TKIs to contribute to advancements in molecular genomics and the treatment of lung cancer.

## 1. Introduction

According to the latest cancer statistics, lung cancer is the leading cause of cancer related death in the United States [1]. Therefore, research aimed at finding effective treatments for lung cancer is prominent.

Lung cancer is divided into two main categories: non-small-cell lung cancer (NSCLC) and small-cell lung cancer (SCLC). NSCLC accounts for approximately 85% of all lung cancer cases. It further divides into three major histological subtypes: adenocarcinoma, squamous cell carcinoma, and large cell carcinoma [2].

Current treatment options for NSCLC include surgical resection, radiation therapy, chemotherapy, immunotherapy, and targeted therapy. Among these, targeted therapy stands out for its ability to precisely target specific proteins and genetic changes involved in cancer growth, resulting in greater effectiveness and fewer side effects compared to traditional treatments [3]. This has sparked significant interest among researchers in understanding the DNA mutations that drive cancer progression. Gaining deeper insights into these molecular mechanisms will enable the development of more effective targeted treatments for lung cancer.

A common genomic characteristic that drives NSCLC is mutations in the epidermal growth factor receptor (EGFR). The EGFR is a tyrosine kinase receptor found in the plasma membrane [4]. Upon binding with its ligand, the EGFR becomes activated, initiating a signaling cascade that promotes cell proliferation, division, and survival [5]. However, mutations in the EGFR gene can result in the receptor being perpetually active, continuously triggering this signaling cascade. This sustained activation significantly enhances cell growth and division, ultimately contributing to the development and progression of cancer.

The most common EGFR mutations associated with NSCLC are the deletion of exon 19 and the L858R mutation in exon 21, which together account for approximately 90% of cases [6,7]. Research shows that about 15% of Caucasian and 50% of Asian NSCLC patients harbor these mutations [8]. Targeted therapies aimed at these specific EGFR mutations have significantly improved survival rates for NSCLC patients, extending them from just over one year to around three years compared to standard chemotherapy [9].

Our paper aims to provide a comprehensive review of the current literature surrounding EGFR TKI targeted therapy for NSCLC. We will provide a history of the various generations of EGFR TKI targeted drugs and explore their clinical efficacy. Additionally, we will discuss emerging resistance mechanisms that are shaping the future of EGFR-targeted treatment. By synthesizing these findings, we hope to offer valuable insights into the optimal use of EGFR TKIs and identify potential areas for further research in the pursuit of a better understanding of the molecular genomics that drive lung cancer and more effective therapies for NSCLC patients.

## 2. Materials and Methods

To gain a comprehensive understanding of the current landscape of EGFR-targeted therapies, we conducted an extensive PubMed search utilizing various combinations of key terms, including “EGFR”, “Epidermal Growth Factor Receptor”, “Tyrosine Kinase Inhibitor”, “TKI”, “Neoadjuvant”, “Adjuvant”, “Locally Advanced Stage”, “Chemotherapy”, “Clinical Outcome”, and “Overall Survival”. This systematic approach allowed us to capture a wide array of research related to EGFR-targeted treatments across different therapeutic contexts.

In this paper, we summarize the major findings related to neoadjuvant and adjuvant therapies, as well as treatments for locally advanced and metastatic NSCLC, which are summarized in Table 1. By analyzing recent studies and clinical outcomes, we aim to highlight the advancements in EGFR-targeted therapies, their impact on treatment protocols, and the overall survival rates for patients. This synthesis not only underscores the evolving strategies in managing EGFR-mutant NSCLC but also identifies potential areas for future research and development. Through this overview, we hope to provide valuable insights that can inform clinical practice and guide ongoing investigations in the field.

## 3. Generations of EGFR TKI Drugs

There are currently three generations of EGFR-targeted therapies used to treat NSCLC; first-, second-, and third-generation EGFR TKIs are designed to interrupt the continuous signaling pathway activated by constitutively active EGFRs. Each generation introduces advancements in targeting efficacy and resistance management, reflecting the ongoing evolution of treatment strategies aimed at improving patient outcomes in treating NSCLC.

### 3.1. First-Generation

The first generation of EGFR inhibitors consists of small TKIs that act as reversible competitive inhibitors of adenosine triphosphate (ATP) to the tyrosine kinase domain [9]. This binding decreases the EGFR’s affinity to ATP and helps block signal transduction, ultimately halting cell proliferation.

**Figure 1:**
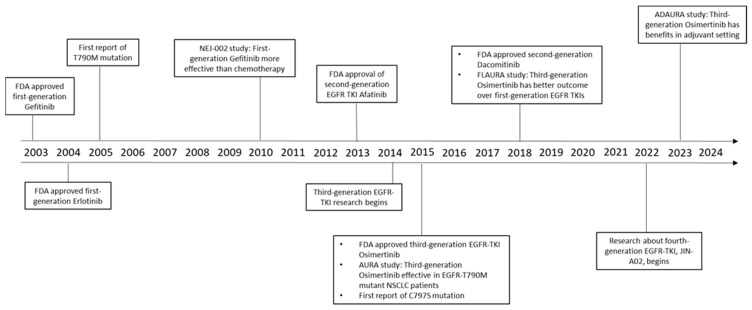
Timeline of developments and major research breakthroughs of first-, second-, third- and fourth-generation EGFR TKIs.

In 2003, the United States Food and Drug Administration (FDA) approved gefitinib as the first clinically approved anti-EGFR therapeutic for patients with NSCLC [20], (Figure 1). This marked a significant milestone in targeted cancer therapy. Following gefitinib’s success, other EGFR inhibitors, such as erlotinib, lapatinib, and icotinib were introduced into clinical practice [21,22,23].

However, first-generation EGFR TKIs soon faced challenges related to drug resistance due to their reversible action, particularly with the emergence of the T790M mutation [24]. Patients treated with gefitinib often experienced disease progression after 9 to 15 months of progression-free survival (PFS), highlighting the limitations of these early therapies and the urgent need for more effective treatment options [25].

### 3.2. Second-Generation

Second-generation EGFR inhibitors, such as afatinib, dacomitinib, and neratinib, were designed as improvements over first-generation agents [26,27,28]. While they share a similar structural framework with their predecessors, these inhibitors are irreversible and possess a unique ability to bind to the C797 residue site through a Michael addition mechanism [29]. This binding effectively inhibits the autophosphorylation of the EGFR, resulting in a more sustained blockade of the signaling pathways associated with tumor growth.

Both first- and second-generation EGFR TKIs achieve relatively high objective response rates, typically in the range of 60–70% [30]. However, significant challenges remain, as the T790M mutation continues to cause many patients to be resistant to these therapies. Furthermore, second-generation inhibitors often exhibit poor selectivity for wild-type EGFRs, leading to a range of adverse reactions, including diarrhea and skin rashes [30,31].

### 3.3. Third-Generation

The first third-generation EGFR TKI was discovered by screening a library of irreversible kinase inhibitors for ones that specifically target the EGFR T790M mutation and can bind to the C797 residue [32]. Osimertinib, which received FDA approval in 2015, is one of the most notable third-generation TKIs [33], (Figure 1). It has demonstrated remarkable efficacy in its clinical trials involving patients with EGFR-positive NSCLC who possess dual mutations, such as the L858R/T790M or ex19del/T790M. These trials also demonstrated that Osimertinib displays limited adverse events, making it a preferred option for many patients [15]. Following the success of Osimertinib, several other EGFR-targeted therapies have emerged. Notable among them are almonertinib, vandetanib, olmutinib, fumonertinib, brigatinib, simotinib, pyrotinib, and mobocertinib [31,34,35,36,37,38].

### 3.4. Fourth-Generation

The development of fourth-generation EGFR TKIs is currently underway to address the mutations and resistance mechanisms that third-generation drugs cannot overcome (Figure 1). Osimertinib has been associated with the emergence of the C797S mutation in about 20% of patients [39]. At present, there are no approved treatments specifically designed to combat the C797S mutation, highlighting a critical gap in available options for patients who experience this form of resistance.

In 2022, JIN-A02 was introduced as a novel fourth-generation TKI [40]. This orally administered compound has shown promising anti-tumor activity in preclinical models, indicating its potential to effectively target the C797S mutation. As research progresses, the development of JIN-A02 and similar agents may greatly improve our capacity to overcome resistance to EGFR-targeted therapies. Ongoing clinical trials will be essential for assessing the efficacy and safety of these innovative compounds in human subjects. 

## 4. EGFR TKIs in a Neoadjuvant Setting

Neoadjuvant therapy, which is administered prior to surgery, is commonly used for operable and locally advanced lung cancer [41]. In patients with stage I or II NSCLC, the primary goal is to eliminate micrometastases, which are small clusters of cancer cells, to improve survival rates [42]. For those with locally advanced stage IIIA NSCLC, neoadjuvant therapy can help downstage tumors, making them more suitable for surgical intervention. However, despite its advantages, neoadjuvant therapy also has drawbacks, such as prolonged tumor presence, delays in surgery, and increased surgical complexity due to thoracic adhesions and fibrosis [41]. Therefore, developing the most effective neoadjuvant therapies is crucial. Particularly of interest is comparing traditional chemotherapy with targeted therapies for patients with EGFR-positive NSCLC.

An open-label phase II study in the Netherlands investigated the safety of neoadjuvant erlotinib treatment in patients with early-stage (TI-3 N0-1) resectable NSCLC [43]. The study included 60 patients who received neoadjuvant erlotinib 150 mg daily for three weeks followed by surgical resection of the tumor. Treatment response was assessed by positron emission tomography (PET) and computed tomography (CT) scans. Seven patients discontinued treatment early, but the results showed that the toxicity was generally mild. This study proved that neoadjuvant erlotinib is safe for use and can be tested further.

EMERGING-CTONG 1103 was the first multicenter trial to compare erlotinib with traditional chemotherapy in a neoadjuvant setting [10]. This open-label phase II randomized study included 72 patients with stage IIIA-N2 NSCLC. In the experimental arm, patients received erlotinib at a dose of 150 mg daily for 42 days, followed by up to 12 months of postoperative therapy. The control arm consisted of patients who underwent two cycles of gemcitabine and cisplatin, followed by up to two cycles of postoperative therapy. Researchers measured the overall response rate (ORR), defined as the percentage of patients whose tumors showed a significant reduction in size after treatment; progression-free survival (PFS), which is the time from the start of the treatment until disease progression or death; and overall survival (OS). Results indicated an ORR of 54.1% in the erlotinib group compared to 34.3% in the chemotherapy group. Additionally, there was a statistically significant difference in median PFS with erlotinib showing 21.5 months versus 11.4 months for chemotherapy. However, OS rates were not statistically significant between the two arms. The erlotinib group had a 3-year OS rate of 58.6% and a 5-year OS rate of 40.8%, while the chemotherapy group reported 3-year and 5-year OS rates of 55.9% and 27.6%, respectively. The differences may be influenced by the additional lines of treatment that patients received. Furthermore, the lack of significant differences in OS could be linked to the use of first-generation EGFR TKIs and the variability in the duration of postoperative adjuvant therapy, which was determined by the investigators. Overall, the findings of this trial emphasize the potential of erlotinib as an effective neoadjuvant treatment option, while also highlighting the need for further research to optimize therapeutic strategies.

In 2022, the Checkmate-816 study revolutionized the field of neoadjuvant therapy for NSCLC by demonstrating the substantial benefits of combining chemotherapy with the immune checkpoint inhibitor nivolumab [11]. The results showed that this combination significantly enhanced survival rates compared to platinum-based chemotherapy alone in patients with stage IB to IIA resectable NSCLC. Notably, the median event-free survival improved from 20.8 months with chemotherapy alone to 31.6 months with the addition of nivolumab. While the combination therapy showed promising efficacy, it also presented treatment-related challenges. Grade three or four adverse events occurred in 33.5% of patients receiving the combination therapy, compared to 36.9% in the chemotherapy-only group, indicating a comparable safety profile. These findings underscore the potential of incorporating targeted therapies such as nivolumab into neoadjuvant treatment regimens, potentially leading to improved clinical outcomes for patients.

Since the Checkmate-816 trial, there has been a notable increase in clinical trials focusing on preoperative targeted therapies, driven by the anticipation of enhanced patient outcomes. Researchers are now exploring various combinations and treatment regimens that integrate immune checkpoint inhibitors and targeted therapies to maximize therapeutic efficacy while minimizing adverse events. This shift in focus represents a significant advancement in the approach to treating resectable NSCLC, ultimately aiming to provide patients with more effective and safer treatment options in the neoadjuvant setting.

One noteworthy ongoing study, NeoADURA, is the first phase III multicenter clinical trial specifically investigating neoadjuvant therapy for patients with resectable stage II to IIIB N2 NSCLC with EGFR mutations [44]. The primary objective of this study is to evaluate the efficacy of neoadjuvant Osimertinib, either as a standalone treatment or in combination with chemotherapy, compared to standard neoadjuvant chemotherapy alone. Participants in the trial are randomly assigned to one of three treatment groups: a placebo combined with the investigator’s choice of platinum-based chemotherapy, Osimertinib at a dose of 80 mg paired with the investigator’s chosen chemotherapy regimen, or Osimertinib as a standalone treatment. The anticipated results from NeoADURA aim to enhance our understanding of the effectiveness of neoadjuvant Osimertinib and to inform future treatment protocols.

## 5. EGFR TKIs in an Adjuvant Setting

Approximately 30% of patients with NSCLC present with resectable disease [45]. The current standard of care for patients with resectable stage II-IIIA NSCLC is surgery followed by adjuvant cisplatin-based chemotherapy. However, the overall survival rate in these patients remains poor, estimated to be about 36% and 49% with a median survival time of 35–58.9 months according to the International Association for the Study of Lung Cancer [46]. A retrospective study from Japan evaluated patients with resected stage II-III adenocarcinoma undergoing adjuvant chemotherapy [47]. The results revealed that platinum chemotherapy did not significantly improve the disease-free survival in EGFR-wildtype or EGFR-mutant populations. However, the OS rate of EGFR-wildtype populations significantly benefited from adjuvant chemotherapy.

### 5.1. Gefitinib vs. Chemotherapy

Based on these findings, the effectiveness of adjuvant EGFR TKIs in patients with stage IIB or IV completely resected advanced NSCLC is gaining attention. In 2017, the CTONG1104 trial assessed this approach with 222 patients in China who had undergone resection for stage II to IIIA NSCLC with EGFR-positive mutations [12]. Participants were randomly assigned to receive either 2 years of gefitinib or 4 cycles of a chemotherapy regimen consisting of cisplatin and vinorelbine. The results showed a statistically significant improvement in median disease-free survival (mDFS) for the gefitinib group, with a mDFS of 30.8 months compared to 19.8 months for those receiving cisplatin plus vinorelbine. This finding highlights the potential of targeted therapies to delay disease recurrence in this patient population. However, it is important to note that the median overall survival did not significantly differ between the gefitinib and vinorelbine groups, suggesting that while gefitinib may enhance early disease control, it does not necessarily lead to longer overall survival at this stage. These results underscore the critical role of targeted therapy as an effective adjuvant treatment option, particularly in improving disease-free survival for patients with EGFR-positive NSCLC.

The IMPACT trial was a randomized study involving 232 patients with resected EGFR-mutated stage II to IIIA NSCLC [13]. Participants were assigned to receive either 2 years of gefitinib therapy or 4 cycles of standard adjuvant chemotherapy. The results revealed that the mDFS was significantly longer for the gefitinib group, at 35.9 months, compared to 25.1 months for the chemotherapy group. However, Kaplan–Meier curves showed that the survival benefits of the two treatments converged around 4 years post-surgery, indicating no statistically significant difference in DFS after this point. Additionally, OS rates at 5 years were comparable, with 78% for the gefitinib group and 74% for the chemotherapy group. While adjuvant gefitinib proved effective in delaying early relapse, the trial concluded that it did not significantly extend DFS or OS compared to traditional chemotherapy. Future studies should explore therapy combinations or different dosing strategies to improve long-term outcomes for patients with EGFR-mutant NSCLC.

### 5.2. Icotinib vs. Chemotherapy

Icotinib has demonstrated comparable efficacy to gefitinib while offering better tolerability for patients with advanced NSCLC [48]. This improved tolerability is especially important, as it can greatly enhance a patient’s quality of life during treatment. Two retrospective studies indicate that icotinib may be beneficial for patients following complete tumor resection, suggesting its potential role in both adjuvant and advanced treatment settings [49,50]

The EVIDENCE trial was a phase III study designed to assess whether the adjuvant use of icotinib could improve clinical outcomes compared to standard adjuvant chemotherapy in patients with EGFR-mutant stage II-IIIA NSCLC [14]. This trial enrolled 322 patients, with 151 assigned to receive icotinib and 132 to standard chemotherapy. The primary endpoint of the study was DFS. Results revealed a 3-year DFS rate of 63.3% for the icotinib group, significantly surpassing the 32.5% rate observed in the chemotherapy group. This difference indicates a statistically significant improvement in survival for patients treated with icotinib, revealing its potential as an effective adjuvant therapy. While OS data are still preliminary, with 14 deaths (9%) reported in the icotinib group and 14 deaths (11%) in the chemotherapy group, these findings lay the groundwork for further investigation into icotinib’s long-term benefits. The EVIDENCE trial highlights the promising role of icotinib in managing EGFR-mutant NSCLC but also suggests the need for additional studies to fully understand its impact on overall survival and to refine treatment strategies for this patient population.

ADAURA was a groundbreaking international study evaluating the efficacy and safety of a third-generation EGFR TKI, Osimertinib, as an adjuvant therapy for patients with completely resected EGFR mutation-positive stage IB to IIIA NSCLC [15]. This double-blind, phase III trial involved 682 patients who were randomized to receive either Osimertinib or a placebo for up to three years, until disease progression or unacceptable toxicity occurred. Postoperative adjuvant cisplatin-based chemotherapy is typically recommended for patients with completely resected stage II to IIIA disease, and selectively for those with stage IB disease. Consequently, ADAURA stratified the patient population by disease stage (IB, II, or IIIA).

The results revealed that in patients with stage IIB to IIIA, EGFR mutation-positive NSCLC, disease-free survival at 24 months was significantly longer for those receiving Osimertinib (90%) compared to the placebo group (44%). Furthermore, Osimertinib was shown to reduce the risk of death by 51%, with a hazard ratio of 0.49. In the overall population, 89% of patients in the Osimertinib group and 85% in the placebo group were alive and free of central nervous system disease. These findings highlight a substantial reduction in the risk of disease recurrence with Osimertinib.

## 6. EGFR TKIs in Locally Advanced or Metastatic Cancer

According to the 8th edition of the TNM staging system, locally advanced NSCLC is classified as stage III disease [51]. Patients diagnosed with this stage typically undergo a standard treatment regimen that includes a combination of chemotherapy followed by the immunotherapy agent durvalumab [52]. This approach has emerged as the current standard of care due to its proven effectiveness in enhancing survival and improving overall outcomes for these patients. In cases where patients present with CNS metastasis, additional local therapies are considered, including surgical interventions, stereotactic radiosurgery, or whole-brain radiotherapy, aimed at managing symptoms and controlling disease progression [53]. However, with the increasing availability of targeted therapies, particularly EGFR TKIs, there is a growing interest in research that evaluates the efficacy of targeted treatments in comparison to traditional options.

The ARUA3 study compared the EGFR TKI Osimertinib and platinum-based chemotherapy in 419 patients with locally advanced NSCLC and the EGFR T790M mutation [16]. Participants were randomly assigned in a 2:1 ratio to receive either Osimertinib or chemotherapy. The results demonstrated that Osimertinib outperformed traditional chemotherapy, as patients treated with Osimertinib experienced a longer median PFS of 10.1 months, compared to just 4.4 months for those receiving chemotherapy. Moreover, for patients with CNS metastases, the benefits of Osimertinib were even more pronounced, with a median PFS of 8.5 months versus 4.2 months for those on chemotherapy. The incidence of grade three or higher adverse events was significantly lower in the Osimertinib group (23%) compared to the chemotherapy group (47%). These findings reveal the potential of Osimertinib as a more effective and better-tolerated treatment option for patients with advanced NSCLC.

The FLAURA study investigated the efficacy of Osimertinib in patients with EGFR T790M mutation-positive advanced NSCLC by comparing it to other standard EGFR TKIs, specifically gefitinib and erlotinib [17]. This double-blind, phase three trial randomly assigned participants to receive either Osimertinib at a dose of 80 mg per day or one of the standard EGFR TKIs: gefitinib at 250 mg per day or erlotinib at 150 mg per day. The results showed that patients treated with Osimertinib experienced a significantly longer median PFS of 18.9 months, in comparison to 10.2 months for those receiving gefitinib or erlotinib. While the ORR and OS were comparable between the groups, the tolerability profile of Osimertinib was notably better. Adverse events of grade three or higher occurred in only 34% of patients receiving Osimertinib, in contrast to 45% in the group treated with standard EGFR TKIs. These findings highlight Osimertinib’s potential as a more effective and better-tolerated treatment option for patients with advanced EGFR-mutant NSCLC.

Aumolertinib (formerly known as HS-10296) is a recently approved third-generation EGFR TKI developed by Hansoh Pharmaceutical Group Co., Ltd. (Shanghai, China). It was evaluated for its efficacy in the double-blind phase III AENEAS study [18]. In this trial, 429 patients with locally advanced or metastatic EGFR-mutant positive NSCLC were randomly assigned to receive either 110 mg of Aumolertinib daily or 250 mg of gefitinib daily. The results demonstrated that patients treated with Aumolertinib experienced a significantly longer median PFS of 19.3 months than those who received gefitinib, who had a median PFS of 9.9 months. While the ORR, disease control rate, and incidence of adverse events were similar between the two groups, the enhanced PFS associated with Aumolertinib highlights its potential as an effective treatment option for this patient population. These findings show the promise of Aumolertinib in advancing therapeutic strategies for patients with advanced EGFR-mutant NSCLC.

A recent breakthrough in the treatment of NSCLC is Fumonertinib (AST2818), a third-generation EGFR TKI developed Allist Pharmaceuticals Co., Ltd of Shanghai, China. Fumonertinib was evaluated in the FURLONG study, a randomized phase III trial that compared it to gefitinib [19]. Participants in the study included adults aged 18 and older who were diagnosed with locally advanced or metastatic NSCLC, specifically those with stage IIIB, IIIC, or IV disease, or with unresectable tumors harboring EGFR exon 19 deletions or the exon 21 L858R mutation. Patients were stratified based on their EGFR mutation status and the presence of central nervous system (CNS) metastases. Of the qualified participants, 178 patients were randomly assigned to receive oral Fumonertinib at a dose of 80 mg per day, while 179 patients received oral gefitinib at a dose of 250 mg per day, both administered in 21-day cycles until disease progression resumed, intolerable toxicities arose, consent was withdrawn, or other reasons for discontinuation occurred, as determined by the investigators. The median progression-free survival (PFS) for the Fumonertinib group was 20 months, significantly longer than the median PFS of 11 months observed in the gefitinib group. These results align with findings from the FLAURA and AENEAS studies, indicating that Fumonertinib shows promise as an effective first-line therapy for Chinese patients with EGFR mutation-positive NSCLC. This suggests that Fumonertinib is a valuable new treatment option for this specific patient population.

## 7. Advancements in Lung Cancer Treatment

In addition to the advances in EGFR TKI drugs, there is increasing focus on creating personalized treatment plans through precision medicine, which considers a patient’s genetics, lifestyle, and environmental factors to identify the most effective therapeutic strategies [54]. A key component of precision medicine is the understanding of biomarkers, which serve as indicators of disease progression and treatment response. Molecular profiles, which include EGFR genes, can help define specific mutations in individuals and are crucial in progressing the treatment of NSCLC.

Recent advancements in big data and artificial intelligence (AI) have significantly enhanced our ability to analyze and interpret these biomarkers. By leveraging large-scale health datasets, AI can identify new relationships between biomarkers and NSCLC, revealing insights that were previously difficult to uncover [55,56,57]. This combination of molecular profiling and cutting-edge technology not only facilitates a deeper understanding of the disease but also paves the way for more targeted and effective treatments, ultimately improving patient outcomes in NSCLC.

## 8. Conclusions

With the advent of EGFR-mutant targeted therapies, a growing body of research has investigated their efficacy across various clinical settings, including neoadjuvant, adjuvant, locally advanced, and metastatic settings. These studies have consistently demonstrated significant advantages of EGFR-targeted therapies over traditional chemotherapy, particularly in terms of improved response rates and potential survival benefits. For example, patients receiving these targeted treatments often experience longer PFS, fewer side effects, and overall better quality of life.

Overcoming cancer resistance in drug development is crucial for advancing effective treatments and improving patient outcomes. Resistance mechanisms can limit the effectiveness of existing therapies, leading to treatment failures and poor prognoses for patients. Addressing these challenges is essential to unlocking the full potential of new and innovative cancer treatments. As ongoing and future research unfolds, it will address several critical questions that remain unanswered. Key areas of focus will include identifying optimal treatment combinations that maximize efficacy while minimizing toxicity, assessing long-term outcomes in diverse patient populations, and evaluating the overall impact of these therapies on quality of life and daily functioning. Furthermore, as we deepen our understanding of EGFR mutations and their intricate roles in tumor biology, we can expect the development of increasingly tailored therapeutic approaches that enhance treatment efficacy and improvement of patient care.

## Figures and Tables

**Table 1:** Summary of studies comparing first-, second-, and third-generation EGFR TKIs with chemotherapy in different settings and their major findings.

Name of Study	Study Design	Intervention Arms	Major Findings:
EMERGING-CTONG1103 [10]	Randomized, phase II study, open-label, Neoadjuvant setting	Erlotinib vs. gemcitabine/cisplatin	Erlotinib arm had significant increase in PFS
Checkmate-816 [11]	Randomized, phase II study, open-label, Neoadjuvant setting	Nivolumab plus chemotherapy vs. chemotherapy alone	Combination therapy more effective than chemotherapy alone
CTONG1104 [12]	Randomized, phase III, Adjuvant setting	Gefitinib vs. chemotherapy	Gefitinib significantly improved DFS
IMPACT [13]	Randomized, phase III, open-label, Adjuvant setting	Gefitinib vs. chemotherapy	Gefitinib significantly improved DFS
EVIDENCE [14]	Randomized, phase III, open-label, Adjuvant setting	Icotinib vs. chemotherapy	Icotinib significantly improved DFS
ADAURA [15]	Randomized, phase III, Adjuvant setting	Osimertinib vs. placebo	Osimertinib significantly improved DFS
ARUA3 [16]	Randomized, phase III, open-label, Locally advanced setting	Osimertinib vs. chemotherapy	Osimertinib arm had significant increase in PFS
FLAURA [17]	Randomized, phase III, Locally advanced setting	Osimertinib vs. gefitinib or erlotinib	Osimertinib more effective than other EGFR TKIs
AENEAS [18]	Randomized, phase III, Locally advanced/metastatic setting	Aumolertinib vs. gefitinib	Aumolertinib significantly increased PFS
FURLONG [19]	Randomized, phase III, Locally advanced/metastatic setting	Fumonertinib vs. gefitinib	Fumonertinib significantly increased PFS
